# Quantitative measurement of odor detection thresholds using an air dilution olfactometer, and association with genetic variants in a sample of diverse ancestry

**DOI:** 10.7717/peerj.643

**Published:** 2014-11-06

**Authors:** Gillian R. Cook, S Krithika, Melissa Edwards, Paula Kavanagh, Esteban J. Parra

**Affiliations:** Department of Anthropology, University of Toronto at Mississauga, ON, Canada

**Keywords:** Scentroid SM110C, Olfactory detection threshold, General olfactory sensitivity (GOS), Genetic association, Genetic marker

## Abstract

Genetic association studies require a quantitative and reliable method for odor threshold assessment in order to examine the contribution of genetic variants to complex olfactory phenotypes. Our main goal was to assess the feasibility of a portable Scentroid air dilution olfactometer for use in such studies. Using the Scentroid SM110C and the SK5 n-butanol Sensitivity Kit (IDES Canada Inc.), n-butanol odor thresholds were determined for 182 individuals of diverse ancestry (mean age: 20.4 ± 2.5 years; *n* = 128 female; *n* = 54 male). Threshold scores from repeat participants were used to calculate a test–retest reliability coefficient, which was statistically significant (*r* = 0.754, *p* < 0.001, *n* = 29), indicating that the Scentroid provides reliable estimates of odor thresholds. In addition, we performed a preliminary genetic analysis evaluating the potential association of n-butanol odor thresholds to six single-nucleotide polymorphisms (SNPs) putatively involved in general olfactory sensitivity (GOS). The results of multiple linear regression analysis revealed no significant association between the SNPs tested and threshold scores. However, our sample size was relatively small, and our study was only powered to identify genetic markers with strong effects on olfactory sensitivity. Overall, we find that the Scentroid provides reliable quantitative measures of odor detection threshold and is well suited for genetic studies of olfactory sensitivity.

## Introduction

Human scent perception abilities are highly variable. It is well documented that variation exists between individuals and between sexes (Hoover, 2010; Keller et al., 2007). Similarly, several studies have identified inter-population differences in olfactory abilities (Eibenstein et al., 2005; Sorokowska et al., 2013; McRae et al., 2013). Matching the high degree of diversity in scent perception phenotypes, there is a correspondingly large amount of genetic variation in olfactory genes. Ongoing research is attempting to link variation at the genetic level to observed phenotypic variation, gaining a deeper understanding of olfactory genetics, and of the genetic contribution to highly personalized scent perception phenotypes (see Mainland et al., 2014; Schütz et al., 2014; McRae et al., 2013). The majority of studies have focused on the impact of genetic variants in olfactory receptor (OR) genes, although there have also been efforts to examine the potential role of variants in auxiliary olfactory genes (Keydar et al., 2013). In addition, gene expression analysis has shown that inter-individual differences in olfactory abilities may be due to differential expression of functional OR genes in the olfactory mucosa (Verbeurgt et al., 2014).

A major bottleneck in the study of olfactory sensitivity has been a lack of instrumentation for accurately measuring smell. Unlike for vision and hearing, there is no standardized test for olfactory sensitivity in either the clinical or research setting (Eibenstein et al., 2005). Numerous psychophysical methods exist, all of which present olfactory stimuli and allow the test subject to report on their experience (Doty, 2006). These tests of olfactory sensitivity evaluate detection or perception thresholds, as well as odor discrimination, identification, memory, intensity and pleasantness (Doty et al., 1995; Eibenstein et al., 2005). The majority of these tests are qualitative, such as the University of Pennsylvania Smell Identification Test (UPSIT), or are not precise enough for the purposes of genetic association studies (Doty et al., 1995; Kremer, Klimek & Mösges, 1998). The most quantitative measurements are gained through odor threshold testing, where the lowest concentration of an odor that the subject can detect is determined without requiring recognition of the smell (Eibenstein et al., 2005).

While similar in principle, individual threshold tests vary in their design. Common differences include the method of odor delivery, and the order in which dilutions are presented during testing. The most simple odor presentation strategy is the ascending method of limits (AML), whereby the series of diluted odorants is always presented from lowest to highest concentration. With an AML strategy, the threshold is estimated from the transition point at which the subject reports odor detection (Doty, 2006). Threshold tests can be either forced choice or non-forced choice in design. Forced choice tests offer one or more blank stimuli. A non-forced choice design requires presentation of a single odor stimulus, such that the test subject is required to respond whether or not they smell the odor from a single sample without blanks presented for comparison (Doty et al., 1995).

Common threshold tests employed today include the Connecticut Chemosensory Clinical Research Center Test (CCCRC), Sensonic Inc.’s Smell Threshold Test (STT), and a component of the Sniffin’ Sticks test battery (Cain et al., 1983; Doty, 2006; Hummel et al., 1997). These three tests are characterized by the use of liquid odorant dilutions, which are delivered in plastic squeeze bottles or modified felt tip pens. Despite the convenience of liquid dilution, air dilution methods are preferable for evaluating olfactory response. Air dilution olfactometry is more precise than liquid dilution, and circumvents issues of odor intensity loss in volatile aqueous solutions (Eibenstein et al., 2005; Kremer, Klimek & Mösges, 1998). However, preparation and administration is generally more expensive and time consuming for air dilutions than for liquid dilutions. Therefore, a portable, affordable and reliable air dilution olfactory threshold test that can be administered in a short amount of time is needed.

The main goals of our study were to (1) assess the suitability of a portable dynamic olfactometer, the Scentroid SM110C (IDES Canada Inc.), for olfactory threshold studies, and (2) carry out a pilot study to examine the potential association of a selected number of GOS genetic variants and n-butanol thresholds in a sample of diverse ancestry. We evaluated the test–retest reliability of the Scentroid SM110C using n-butanol as an odorant (SK5 n-butanol Sensitivity Kit, IDES Canada Inc). The odorant n-butanol is widely used in olfaction studies, in part due to its variable odor detection threshold (Doty et al., 1995; Hummel et al., 1997). We surveyed the olfactory thresholds for n-butanol in a multi-ethnic population, and genotyped the participants at six missense single-nucleotide polymorphisms (SNPs) located in putative auxiliary olfactory genes. The markers were chosen for their potential involvement in general olfactory sensitivity (GOS), a phenotype characterized by correlation of sensitivities to multiple odorants (Keydar et al., 2013). Genetic variants involved in GOS affect the olfactory thresholds of numerous odorants, making them easier to detect than OR variants when the odor threshold is measured for a single odorant.

We genotyped five SNPs in three genes that had been identified in a database of candidate auxiliary olfactory genes (http://genome.weizmann.ac.il/GOSdb): rs17712299 and rs17712299 in the ATP-binding cassette, sub-family A, member 13 gene (*ABCA13*); rs2889732 and rs13036385 in the BPI fold containing family B, member 4 gene (*BPIFB4*); and rs6746030 in the sodium channel, voltage-gated, type IX, alpha subunit gene (*SCN9A*) (Keydar et al., 2013). The *SCN9A* variant rs6746030 has previously been implicated as having an effect on olfactory function (Heimann et al., 2013; Weiss et al., 2011). In addition to genes identified in the GOS database (GOSdb), we genotyped the SNP rs6265 located in the brain-derived neurotrophic factor gene (*BDNF*), which has been linked to olfactory ability in previous studies (Hedner et al., 2010; Tonacci et al., 2013).

## Materials and Methods

### Subjects

182 participants were recruited on the University of Toronto at Mississauga campus and ranged in age from 18 to 32 years old (mean age: 20.4 ± 2.5 years; *n* = 128 female; *n* = 54 male). 60 individuals were of East Asian ancestry (Chinese, Japanese, Korean, and Taiwanese), 55 were of European ancestry, 58 were of South Asian ancestry (Bangladeshi, Indian, Pakistani, and Sri Lankan), and nine were of mixed or other ancestry. Table 1 describes the main characteristics of the sample. Prior to testing, participants were asked not to eat or drink for twenty minutes. All the individuals gave written informed consent prior to participating in the study. The research protocol was approved by the University of Toronto Research Ethics Board. This study complies with the Declaration of Helsinki for Medical Research involving Human Subjects.

**Table 1:** Sample characteristics by ancestry.

	Total sample	East Asian	European	South Asian	Other
**N (M, F)**	182 (54, 128)	60 (15, 45)	55 (18, 37)	58 (17, 41)	9 (4, 5)
**Mean age ± SD**	20.4 ± 2.5	20.8 ± 2.5	21.0 ± 2.7	19.6 ± 2.1	19.7 ± 1.3

### Olfactory threshold testing

**Figure 1:**
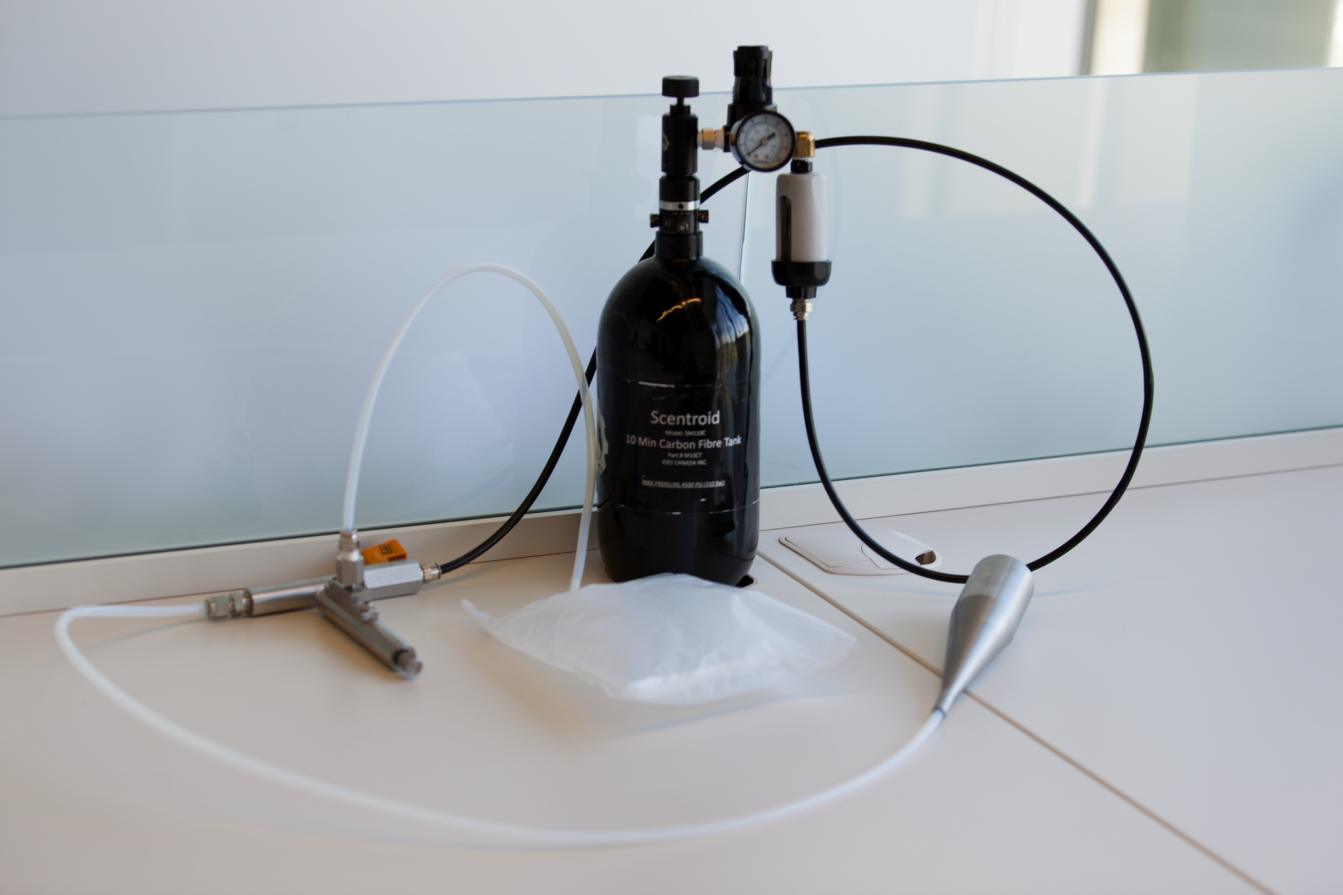
Scentroid SM110C olfactometer used in measuring n-butanol olfactory thresholds.

Olfactory thresholds for n-butanol were assessed using the Scentroid SM110C olfactometer and the SK5 n-butanol Sensitivity Kit (IDES Canada Inc.), shown in Fig. 1. This instrument complies with the European Standard EN13725:2003 for dynamic olfactometers. Additionally, results obtained with the Scentroid SM110 have previously been shown to correlate well with those from established odor assessment techniques. Bokowa (2012) reported very good correlation between the results obtained with the Scentroid SM110 and traditional odor evaluations using a dynamic olfactometer. Szyłak-Szydłowski (2014) reported very strong correlations between the odor concentrations estimated by the Scentroid SM-100 and the Nasal Ranger for Tetrahydrothiophene and Hydrogen sulphide (*r* values 0.98 and 0.93, respectively), and also for field tests on a water treatment plant (*r* = 0.89).

**Figure 2:**
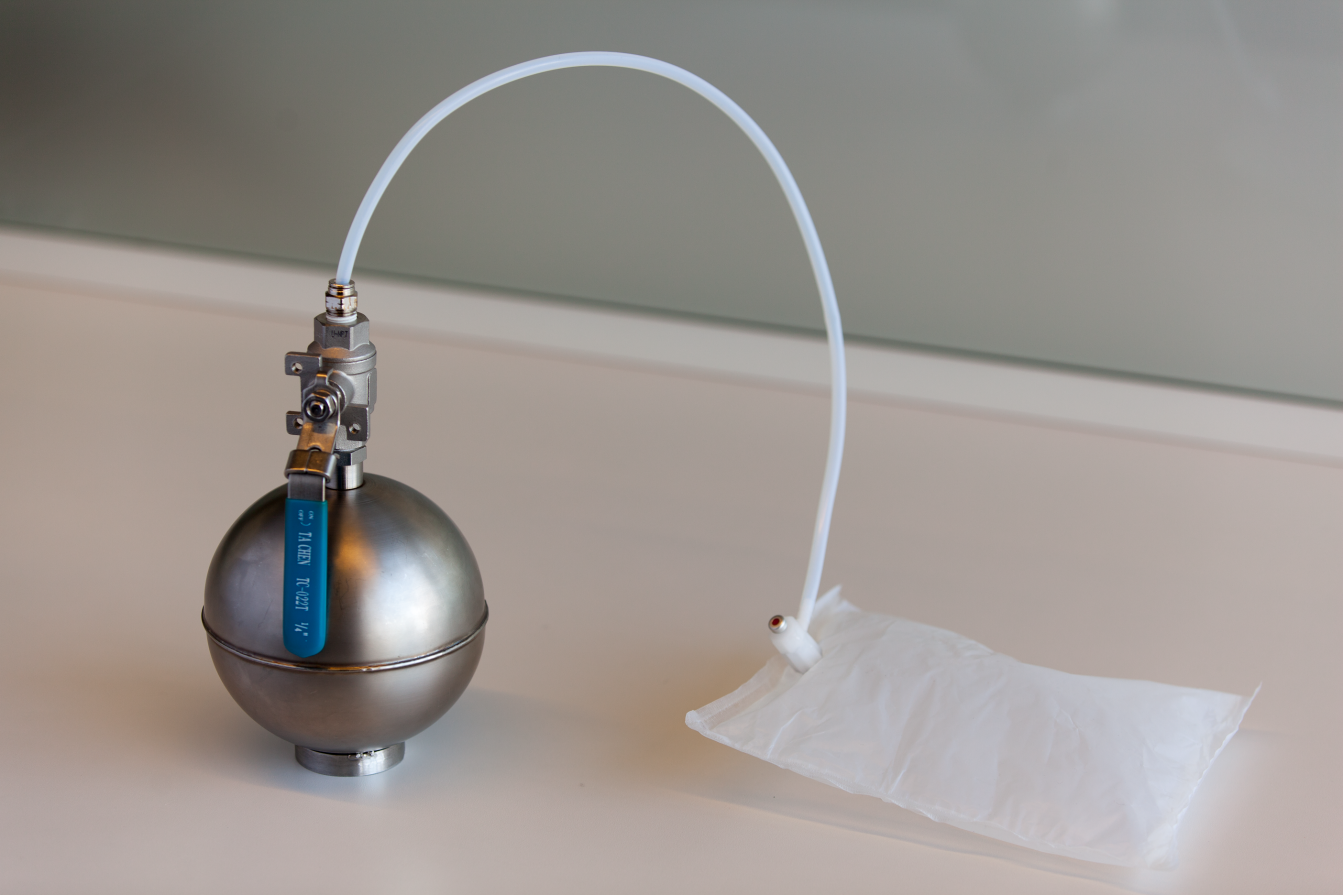
Stainless steel evaporation chamber used for transferring diluted n-butanol to a Tedlar bag.

**Figure 3:**
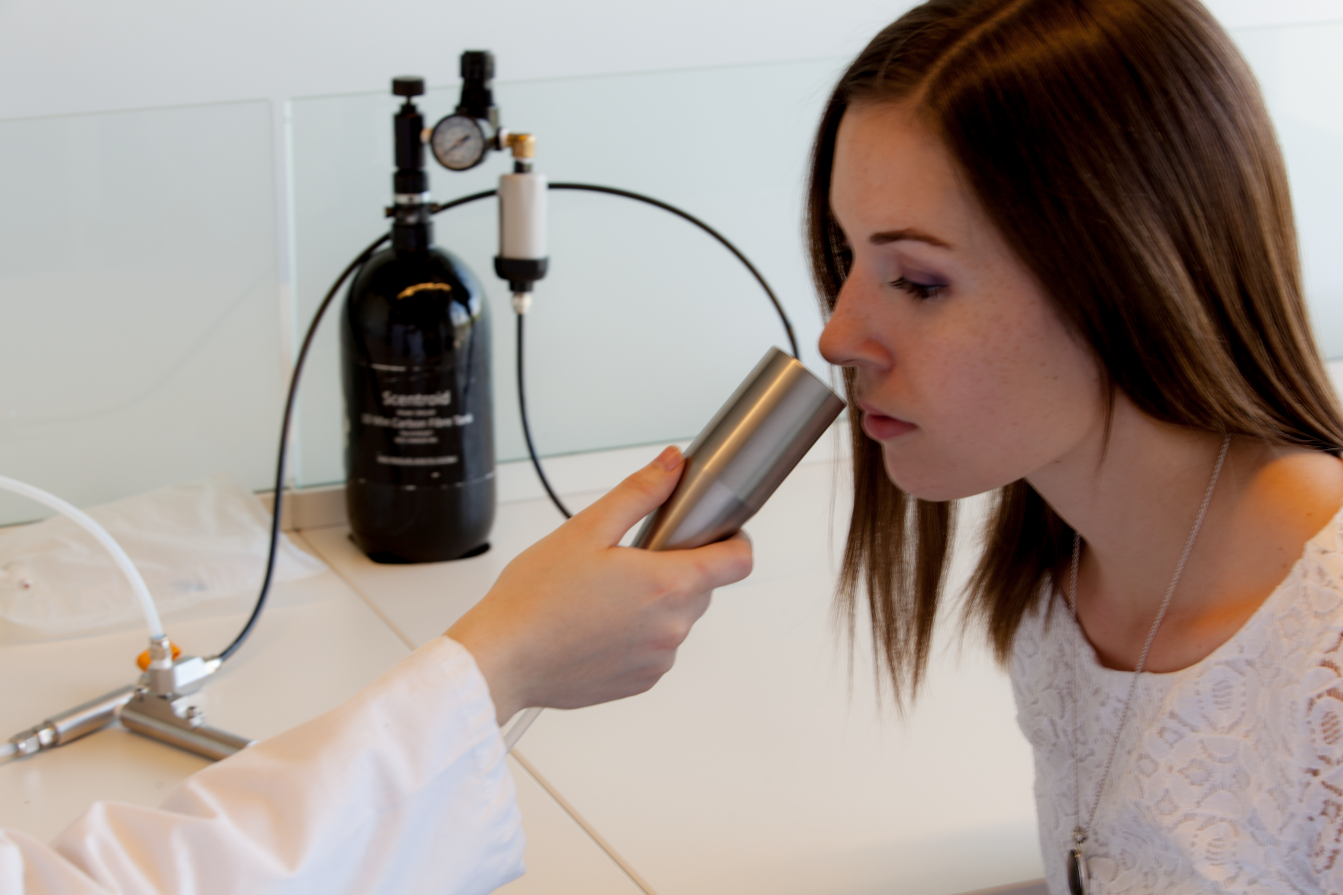
Olfactory threshold testing using the Scentroid SM110C olfactometer and SK5 n-butanol Sensitivity Kit.

The odor was presented birhinally using a non-forced choice strategy that consisted of fifteen steps with dilution ratios ranging from 200 to 3,600. A fresh dilution of n-butanol was prepared daily in a stainless steel evaporation chamber by combining 0.5 µl of aqueous n-butanol with compressed air, equivalent to a concentration of 20 ppm, and was subsequently stored in a 1 L Tedlar bag (Fig. 2). Using the Scentroid’s standardized restriction plate two in the flow regulator allowed for the 20 ppm sample of n-butanol to be further diluted within a 5.5 to 100 ppb range. Subjects were presented with the lowest concentration of n-butanol first and, following an AML strategy, were exposed to successively increased concentrations. A metal cone expelling air at a constant flow of 20 L/min and velocity of 2.5 m/s was presented to each subject for approximately 2 s per dilution step, followed by a brief stimulus break (Fig. 3). Subjects were asked to verbally respond whether or not they could detect the odor at each step. The olfactory threshold was determined from the transition point at which the subjects reported odor detection, and calculated as the mean of the scores from three consecutive tests. Each individual threshold test took approximately 1 to 3 min to complete.

A replication sample consisting of 29 participants was tested 14 to 18 weeks after their original testing date. The olfactory threshold test protocol remained unchanged.

### Genotyping

Saliva was collected with the Oragene DNA OG-500 kit (DNA Genotek Inc.) for genomic DNA extraction following recommended protocols. Samples were sent to LGC Genomics for genotyping by means of the KASP genotyping assay. Genotyping was performed for six SNPs with potential involvement in GOS phenotypes: rs6265, rs2889732, rs6746030, rs13036385, rs17132289, and rs17712299.

### Statistical analyses

The olfactory threshold scores were found to be positively skewed and a log transformation was applied. Using the log-transformed data, an ANOVA was run to examine differences in n-butanol olfactory thresholds between ancestry groups. Test–retest reliability was evaluated using correlation tests and Bland-Altman plots, based on the olfactory threshold scores obtained from a subset of participants who were measured twice within an interval of 14 to 18 weeks. Associations between SNPs and olfactory sensitivity were analyzed through multiple linear regression, with age and sex included as covariates. The association tests were done individually for each ancestry group and also for the full sample, with ancestry as an additional covariate. For the association tests, we used a genotypic model, where the effects of one of the homozygous categories and the heterozygous category are reported in relation to the remaining homozygous category, which is used as a reference. The statistical analyses were carried out with IBM SPSS Statistics v.20. For all analyses the level of significance was set to alpha = 0.05. Genotype data was checked for deviations from Hardy-Weinberg equilibrium, using exact tests available at http://ihg.gsf.de/cgi-bin/hw/hwa1.pl. Post-hoc power analysis was performed with the program Quanto (http://biostats.usc.edu/Quanto.html), using several inheritance models (additive, dominant and recessive) and allele frequencies ranging from 0.05 to 0.50. Parameters were set based on observed n-butanol threshold scores in ppb (mean = 22, SD = 14), with a sample size of 60 and allelic effect sizes ranging from 1 to 10 beta.

## Results

### Analysis of olfactory thresholds in individuals of diverse ancestry

**Figure 4:**
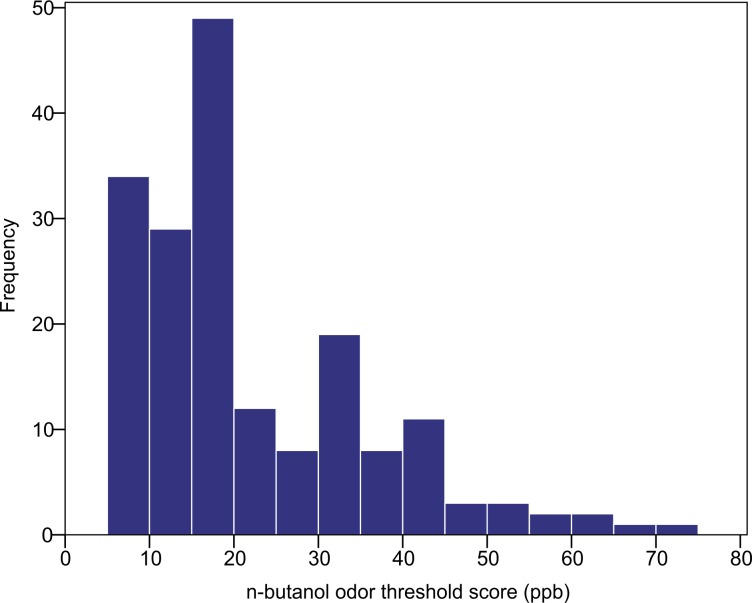
Distribution of n-butanol olfactory threshold scores for 182 participants of diverse ancestry.

The n-butanol olfactory threshold was evaluated in 182 participants of diverse ancestry using the Scentroid SM110C olfactometer. Figure 4 shows the distribution of olfactory threshold in the total sample. The distributions observed in each ancestry group are provided as Fig. S1). The average (±SD) odor threshold concentration in the full sample was 22.5 ± 13.7 ppb. The average concentrations in the European, East Asian and South Asian groups were 20.9 ± 13.0, 24.4 ± 16.2 and 22.1 ± 11.8 ppb, respectively. Odor thresholds were non-normally distributed, and were log-transformed prior to statistical analysis. Using one-way ANOVA, we examined the impact of age, sex and ancestry on log-transformed n-butanol threshold scores. All three factors were not significant (*p* = 0.615 age; *p* = 0.053 sex; *p* = 0.586 ancestry).

**Figure 5:**
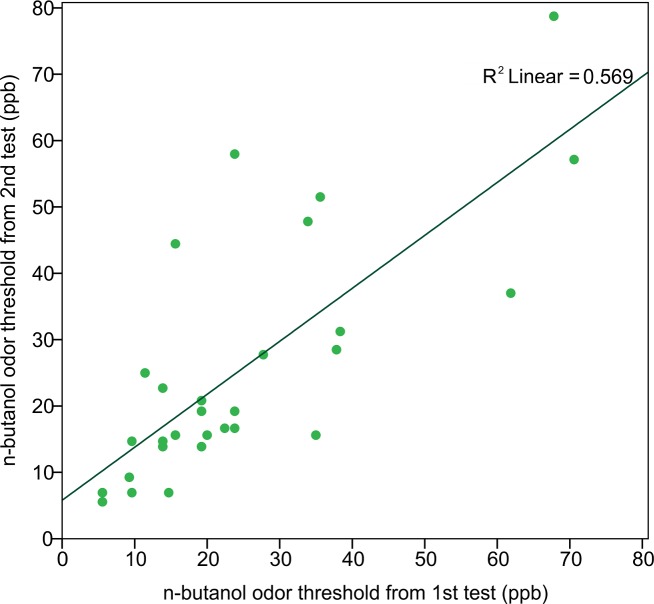
Correlation of n-butanol olfactory threshold scores obtained from participants tested twice, 14 to 18 weeks apart.

**Figure 6:**
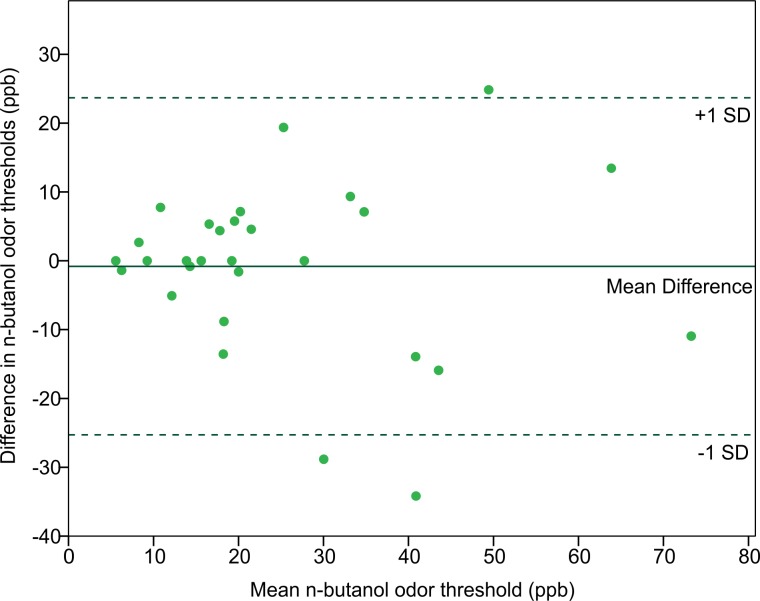
Bland-Altman plot comparing the n-butanol olfactory threshold scores obtained from participants tested twice, 14 to 18 weeks apart.

In order to assess the reliability of the Scentroid olfactometer and our protocol for determining olfactory threshold, the thresholds from returning participants were used to calculate a test–retest reliability coefficient (Pearson correlation coefficient) (Fig. 5). The resulting value of 0.754 was statistically significant (*p* < 0.001; *n* = 29). We also evaluated reliability using Bland-Altman plots, which are shown in Fig. 6. This plot revealed three distinct outliers, whose removal resulted in an improved test–retest reliability coefficient of 0.880. Furthermore, the mean olfactory thresholds (ppb) did not differ significantly between time points, as evidenced by the results of a repeated measures ANOVA (*p* = 0.731, *n* = 29).

### Association of putative GOS SNPs with olfactory thresholds

Six SNPs that have been putatively linked with GOS were genotyped using the KASP assay. The concordance rate observed for blind duplicates was 100%. The genotype distributions of all six SNPs met the assumptions of Hardy-Weinberg equilibrium (*p* > 0.05).

Using multiple regression analysis, each SNP was tested for association with olfactory threshold. The results are depicted in Table 2. The analysis for rs6265, rs2889732, rs6746030, rs13036385, rs17132289 and rs17712299 yielded no significant results, either in the individual ancestry groups or the total sample.

**Table 2:** Results of multiple linear regression analysis using a genotypic model, organized by ancestry. None of the markers are significant at a *p*- value threshold of 0.05.

			East Asian		European		South Asian		Total sample	
SNP	Reference Genotype	Genotype	Beta	*p*- value	Beta	*p*- value	Beta	*p*- value	Beta	*p*- value
rs6265	*TT*	*CC*	0.121	0.453	−0.501	0.256	−0.098	0.698	−0.670	0.596
		*TC*	0.085	0.594	−0.420	0.342	−0.103	0.684	−0.005	0.965
rs2889732	*AA*	*CC*	−0.138	0.702	−0.173	0.208	−0.183	0.270	−0.069	0.525
		*AC*	−0.283	0.431	−0.024	0.876	−0.076	0.640	−0.132	0.202
rs6746030	*GG*	*AA*								
		*GA*	0.078	0.566	0.201	0.141	0.139	0.313	0.120	0.131
rs13036385	*GG*	*CC*	−0.026	0.841	0.025	0.860	−0.064	0.697	0.016	0.846
		*GC*	−0.126	0.340	0.017	0.904	0.097	0.494	−0.016	0.841
rs17132289	*AA*	*TT*	−0.004	0.978					−0.005	0.946
		*AT*	−0.049	0.718	−0.173	0.208			−0.107	0.182
rs17712299	*TT*	*CC*	−0.001	0.994					0.001	0.990
		*TC*	−0.008	0.953	−0.104	0.459	0.222	0.111	0.035	0.654

## Discussion

### Threshold test

We measured the n-butanol olfactory threshold in 182 subjects, in order to assess the feasibility of using the Scentroid SM110C air dilution olfactometer to test olfactory sensitivity. Reliability for this instrument was established using return participant test scores, from which a Pearson correlation coefficient was calculated. The derived test–retest reliability coefficient, representing the linear relationship between the first and second test values, was found to be high (*r* = 0.754, *p* < 0.001). Therefore, the Scentroid air dilution olfactometer, when used in conjunction with a non-forced choice AML strategy, produces reliable n-butanol olfactory thresholds, and compares favorably with many other n-butanol threshold tests (Table 3), for which the correlation coefficients range from 0.26 to 0.86 (Hummel et al., 1997; Linschoten et al., 2001; Lam et al., 2006; Doty et al., 1995).

**Table 3:** Test–retest reliability coefficients of various n-butanol olfactory threshold tests.

Olfactory test	Test-retest reliability coefficient	Sample size	*p*- value	Reference
Connecticut chemosensory Clinical Research Center (CCCRC)	0.36	104	<0.001	(Hummel et al., 1997)
	0.49	57	<0.001	(Doty et al., 1995)
CCCRC modification using ML-PEST	0.26 to 0.86^a^	27	0.190 to <0.001	(Linschoten et al., 2001)
CCCRC done as part of the Combined Olfactory Test (COT)	0.85	35	<0.001	(Lam et al., 2006)
Sniffin’ sticks	0.61	104	<0.001	(Hummel et al., 1997)
Scentroid SM110C	0.76	29	<0.001	

**Notes.**

a Variation in test–retest reliability coefficients due to time between retests.

Currently, the two most common methods for measuring olfactory thresholds are Sniffin’ Sticks, and versions of the CCCRC. The Sniffin’ Sticks olfactory threshold test is applied birhinally, using n-butanol as the odorant in a triple forced-choice strategy using a staircase presentation method. Staircase strategies differ from the AML in the presentation order of the dilution series, and therefore in how thresholds are estimated (see Doty, 2006). Unique to Sniffin’ Stick is the application of felt tipped pens for odor presentation. The felt pens are easy to administer, making this particular test highly popular as a result (Hummel et al., 1997; Kobal et al., 2000). In the CCCRC threshold test, subjects are presented with two plastic squeeze bottles, one containing a blank and the other a set dilution of aqueous n-butanol. The basic procedure is two-alternate forced-choice with an AML presentation of the dilution series. Between nine and 13 dilutions are used, with varying n-butanol concentrations (Cain et al., 1983). Various olfactory tests based on the CCCRC also exist, including the Combined Olfactory Test (COT) in China. It includes both identification and threshold tests, the former of which is culturally adapted. The threshold test component is indistinguishable from that of the CCCRC, yet threshold scores reported in the literature vary (Lam et al., 2006).

Variability in the olfactory threshold scores of individual tests can be attributed to differences in experimental design and the study sample. The age of the subjects tested is a significant factor in evaluation of olfactory sensitivity due to the degradation of the sense of smell with age (Hoover, 2010). The mean age of the individuals who participated in the studies listed in Table 3 ranged from 31.6 to 49.5 years (Linschoten et al., 2001; Hummel et al., 1997). The mean age of our sample is 20.4 years, which is between 10 and 30 years less than those previously cited. In determining a test’s reliability, the time between retests is an important aspect of the research protocol that can impact the results. Our test–retest interval of 14 to 18 weeks is long relative to the studies listed in Table 3, whose intervals range from one day to three weeks, with an average of two weeks. Past research was unable to find support for a relationship between test–retest scores and the time interval between tests (Doty et al., 1995). However, this was based on the examination of relatively short intervals of less than two weeks. Linschoten et al. (2001) included retests performed up to three weeks after initial testing and identified a general trend, whereby the correlation of threshold scores decreased as the test interval increased. This reflects the influence of environmental factors, which act alongside genetics, and impact olfactory sensitivity(Doty, 2006; Hasin-Brumshtein, Lancet & Olender, 2009).

As highlighted previously, the staircase odor presentation strategy of the Sniffin’ Sticks test differs from the AML presentation used in the CCCRC and our protocol. Likewise, the mode of stimulus delivery varies from felt-tipped pens, to plastic bottles, to the Scentroid air dilution olfactometer. Yet, the Scentroid as a method for odor delivery is distinctive. A single sample of the odorant needs to be prepared, and using compressed air, the instrument then further dilutes that stock sample for presentation to the test subject. The test administrator can easily switch between set dilutions, thus altering the compressed air to sample ratio.

Here, we measured n-butanol olfactory thresholds using the Scentroid SM110C air dilution olfactometer. The resulting test–retest reliability coefficient indicates that the Scentroid produces reliable n-butanol olfactory thresholds. While we employed the AML strategy in a non-forced choice manner, adjustments to this protocol could improve the accuracy of odor thresholds obtained with a Scentroid. Multiple-forced choice designs are less subject to response biases, increasing the reliability of resultant threshold scores (Doty et al., 1995). Moreover, the AML odor presentation strategy is less reliable than simple staircase methods, which in turn are associated with higher error rates than the maximum-likelihood adaptive staircase (ML-PEST) (Linschoten et al., 2001). However, such changes in experimental design will increase the test’s administration time and complexity.

### Olfactory SNPs

In addition to testing the reliability of the Scentroid SM110C olfactometer, we carried out a pilot study in which we genotyped six SNPs in genes potentially involved in GOS phenotypes, aiming to investigate genetic associations with n-butanol olfactory thresholds. Our goal was to examine variants that may contribute to overall olfactory sensitivity.

Studies have shown that individuals with heightened or decreased sensitivity to one odor tend to display similar sensitivities to multiple odorants (Cain & Gent, 1991). This has been explained by variation in auxiliary olfactory genes, which are involved in aspects of olfaction other than direct odorant binding, influencing neuronal development or relaying the signal generated by odorant–receptor interaction (Keydar et al., 2013; Hasin-Brumshtein, Lancet & Olender, 2009).

From the GOS database of candidate auxiliary olfactory genes developed by Keydar et al. (2013), we chose SNPs from three genes. Our regression analysis revealed no association between olfactory threshold and genotype for the variants examined in *ABCA13* and *BPIFB4*. There has been no direct evidence of these genes having an olfactory effect in humans. *ABCA13* and *BPIFB4* were included in the GOSdb based on RNA sequences in humans and mice, as well as rat proteome data. Conversely, *SCN9A* has been associated with human olfactory function in two separate studies, in addition to being included in the GOSdb. The voltage-gated sodium channel encoded by *SCN9A* is expressed in both nociceptive and olfactory sensory neurons, and is linked to altered pain and olfactory perception phenotypes. Individuals with loss-of-function mutations in *SCN9A* cannot feel pain, and exhibit general anosmia (Weiss et al., 2011). An investigation of gain-of-function mutations in this ion channel found that haplotypes carrying the minor alleles of the rs6746030 and rs41268673 polymorphisms were associated with increased pain sensitivity and general olfactory acuity (Heimann et al., 2013) with respect to the wild-type haplotypes. In our study, we did not observe a significant effect of this polymorphism in olfactory detection thresholds, either in the full sample or individual ancestry groups.

The Val66Met polymorphism in *BDNF*, rs6265, has been linked to age-related decline in olfactory sensitivity (Hedner et al., 2010). Initially, the effect was only seen among subjects between 70 and 90 years of age. However, a study investigating the effects of Val66Met in younger individuals found an association with olfactory function for a sample with a mean age of 38.7 years (Tonacci et al., 2013). Our study failed to replicate these results, though this may be due to the relatively low mean age of our sample (mean age: 20.4 years).

We performed a small-scale genetic association test for potential GOS variants, utilizing n-butanol olfactory thresholds. No significant results were found in this pilot study. For some variants, this may be due to lack of involvement in GOS. However, several of the SNPs examined have been linked to human olfactory sensitivity previously. Our investigation was hindered by a small sample size, which was further reduced through independent analysis of each ancestry. Our post-hoc power analyses indicate that our study is only adequately powered to identify alleles with strong effects on olfactory sensitivity. For example, assuming an additive model of inheritance (Table S1), we estimated that we have 80% power to identify common alleles (allele frequencies (0.35–0.50)) with effect sizes of 7 ppb or higher. However, our power decreases substantially for alleles with lower allele frequencies and/or smaller effect sizes. Therefore, it is possible that the SNPs examined here have minor effects on inter-individual differences in olfactory sensitivity, and thus should continue to be candidates in future studies.

## Conclusion

In summary, in this study we show that the Scentroid SM110C provides reliable quantitative estimates of odor thresholds, and would be an adequate olfactometer to explore the genetic basis of olfactory sensitivity. Air dynamic olfactometers, which allow a continuous control of the concentration of odorant, have advantages over methods based on liquid dilutions in bottles, which may loose their smell intensity with time (Eibenstein et al., 2005; Kremer, Klimek & Mösges, 1998). A distinct advantage of the Scentroid SM110C with respect to other olfactometers is that it is portable, conferring flexibility in terms of the sampling location. Adequate phenotype characterization is a critical step in the success of genome-wide association (GWA) studies. For traits with an underlying quantitative distribution, using quantitative measures, instead of threshold-based dichotomous outcomes, enhances statistical power because of the larger information content (Plomin, Haworth & Davis, 2009; Smith & Newton-Cheh, 2009). However, it is also important to consider the precision of the estimates, as it has been shown that measurement error reduces statistical power and can have a strong effect on the profile of SNPs identified in GWA studies (Barendse, 2011; Liao et al., 2014). Measurement errors can be reduced by taking multiple measurements (Barendse, 2011; Liao et al., 2014). In our study, we took three measurements per individual, and used the mean value as the dependent variable in our association tests. Using the AML strategy employed in this study, it was possible to get reliable, quantitative measures of odor threshold in less than 5 min per participant. This is particularly important for genome-wide association studies focusing on olfactory sensitivity, which ideally should include thousands of participants in order to identify markers with relatively small effects on olfactory sensitivity. Here, we have primarily focused on odor threshold as a measure of olfactory sensitivity. Other types of test have been developed to evaluate olfactory sensitivity, such as tests for odor discrimination, odor identification, odor memory, and suprathreshold scaling of odor intensity and pleasantness (Doty, 1995). All these tests capture different aspects of olfactory performance.

## Supplemental Information

10.7717/peerj.643/supp-1 Supplemental Information 1 Raw data

10.7717/peerj.643/supp-2 Figure S1 Comparison of n-butanol olfactory threshold score distributions for participants of East Asian, European and South Asian ancestry (n = 60, 55 and 58, respectively)

10.7717/peerj.643/supp-3 Table S1 Power analysis results in a range of beta (ppb) values and allele frequencies for the genetic association testing of SNPs and olfactory sensitivity, assuming an additive model
