# Supplementary figures and images for: Quantitative measurement of odor detection thresholds using an air dilution olfactometer, and association with genetic variants in a sample of diverse ancestry
Source: PeerJ. 2014 Nov 6;2:e643. doi: 10.7717/peerj.643 (PMC4226646; doi:10.7717/peerj.643)

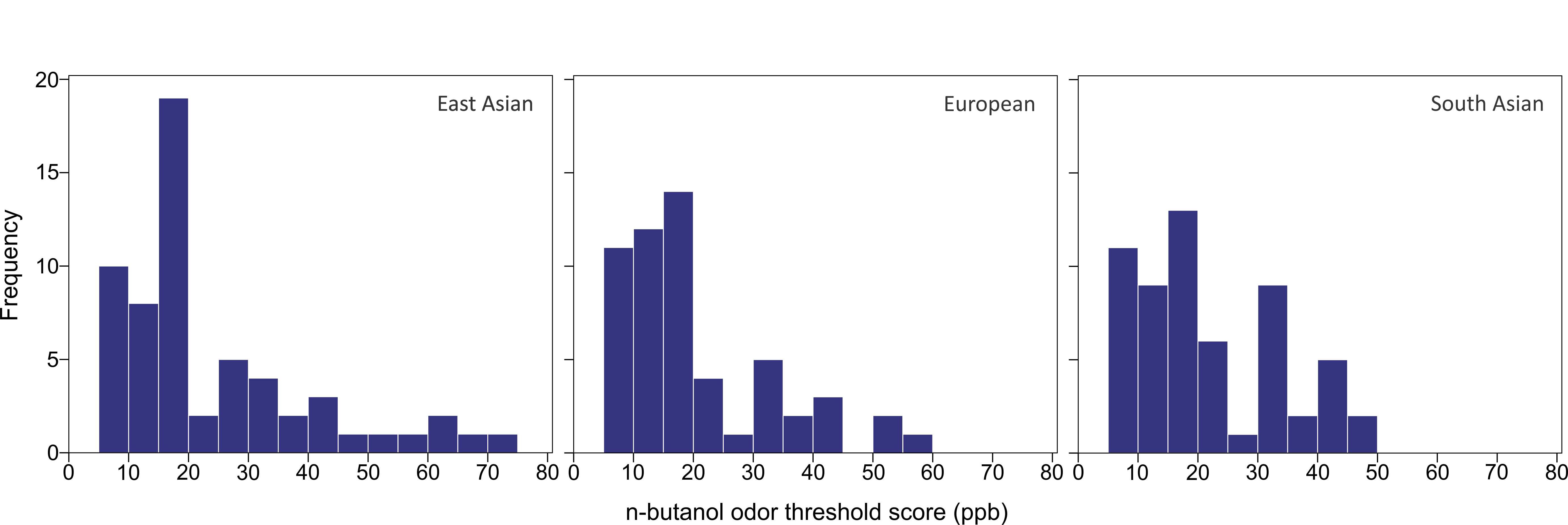

Supplement: Figure S1 [file peerj-02-643-s002.png]
