# Supplementary material for: Quantitative measurement of odor detection thresholds using an air dilution olfactometer, and association with genetic variants in a sample of diverse ancestry
Source: PeerJ. 2014 Nov 6;2:e643. doi: 10.7717/peerj.643 (PMC4226646; doi:10.7717/peerj.643)
Supplement: Table S1 [file peerj-02-643-s003.docx]

Supplementary Table 1. Power analysis results at a range of beta (ppb) values and allele frequencies for the genetic association testing of SNPs and olfactory sensitivity, assuming an additive model.

|  |  | **Beta** | | | | | | | | | |
| --- | --- | --- | --- | --- | --- | --- | --- | --- | --- | --- | --- |
|  |  | **1** | **2** | **3** | **4** | **5** | **6** | **7** | **8** | **9** | **10** |
| **Allele Frequency** |  | **Power (%)** | | | | | | | | | |
| 0.05 |  | 5.3 | 6.3 | 8.1 | 10.5 | 13.7 | 17.7 | 22.5 | 28.0 | 34.1 | 40.8 |
| 0.10 |  | 5.6 | 7.6 | 10.9 | 15.6 | 21.9 | 29.5 | 38.3 | 47.9 | 57.7 | 67.2 |
| 0.15 |  | 5.9 | 8.7 | 13.4 | 20.3 | 29.1 | 39.6 | 51.1 | 62.7 | 73.4 | 82.4 |
| 0.20 |  | 6.1 | 9.6 | 15.6 | 24.3 | 35.3 | 47.9 | 60.9 | 73.0 | 83.1 | 90.5 |
| 0.25 |  | 6.3 | 10.4 | 17.5 | 27.7 | 40.3 | 54.4 | 68.1 | 79.9 | 88.9 | 94.6 |
| 0.30 |  | 6.5 | 11.1 | 19.1 | 30.4 | 44.4 | 59.3 | 73.2 | 84.5 | 92.3 | 96.7 |
| 0.35 |  | 6.6 | 11.6 | 20.3 | 32.6 | 47.4 | 62.9 | 76.8 | 87.4 | 94.2 | 97.8 |
| 0.40 |  | 6.7 | 12.0 | 21.2 | 34.1 | 49.5 | 65.3 | 79.1 | 89.2 | 95.4 | 98.4 |
| 0.45 |  | 6.8 | 12.2 | 21.7 | 35.0 | 50.7 | 66.7 | 80.4 | 90.2 | 96.0 | 98.7 |
| 0.50 |  | 6.8 | 12.3 | 21.9 | 35.3 | 51.1 | 67.2 | 80.8 | 90.5 | 96.1 | 98.8 |
